# Optical Coherence Tomography of Plaque Erosion and Thrombus in Severe Vertebral Artery Stenosis

**DOI:** 10.3390/diagnostics11040638

**Published:** 2021-04-01

**Authors:** Lin Yan, Adam A. Dmytriw, Bin Yang, Liqun Jiao

**Affiliations:** 1 Department of Neurosurgery, Xuanwu Hospital, Capital Medical University, China International Neuroscience Institute (China-INI), Beijing 100053, China; yanlin@xwhosp.org (L.Y.); yangbin@xwhosp.org (B.Y.); 2 Department of Interventional Neuroradiology, Xuanwu Hospital, Capital Medical University, Beijing 100053, China; admytriw@bwh.harvard.edu; 3 Neuroradiology & Neurointervention Service, Brigham and Women’s Hospital, Harvard Medical School, Boston, MA 02115, USA

**Keywords:** vertebral artery stenosis, red thrombus, optical coherence tomography

## Abstract

A 69-year-old male presented with medically refractory vertebrobasilar insufficiency and paroxysmal subjective dizziness for six months. Severe stenosis of a dominant left V2 vertebral artery segment was identified on digital subtraction angiography (DSA) with an irregular intraluminal filling defect immediately above the stenosis. Optical coherence tomography (OCT) demonstrated a normal lumen at the distal end, with red thrombus detected distal to the stenosis. Atherosclerotic plaque containing fibro-lipid was also identified and treated with a drug-eluting stent. Distal red thrombi were not covered by stenting, indicating embolization risk in the future. Clear posterior fossa symptoms occurred after intervention, and treatment with a standard dual antiplatelet regimen and statin therapy was prescribed for one year. Six months after treatment, the symptoms improved, and six-minute walking distances were successful with no gait impairment. To our knowledge, this is the first V2 segment stenosis assessed by OCT imaging before and after stenting, indicating an intact fibrous cap with thrombus formation, as well as plaque erosion. Understanding the role and careful use of OCT may improve the identification of red thrombus and plaque erosion when clinically indicated.

A 69-year-old male presented with medically refractory vertebrobasilar insufficiency and paroxysmal subjective dizziness for six months. The patient had a history of alcohol use and cigarette smoking for more than 40 years, a four-year history of well-controlled essential hypertension, and a one-month history of documented hyperlipemia. No prior stroke, transient ischemic attack (TIA), myocardial infarction, or significant family history was reported. There was no history of anticoagulation or antiplatelet use. The patient presented with an unremarkable neurological examination. Severe stenosis of the dominant left vertebral artery V2 segment was identified on computed tomography angiography (CTA), likely secondary to atherosclerosis, coexisting with an undeveloped left posterior cerebral artery (PCA) and leading to an incomplete circle of Willis, which was noted (Figure 1A–D). Digital subtraction angiography (DSA) demonstrated a corresponding irregular intraluminal filling defect with severe stenosis, and a plan to stent the left V2 stenosis was made (Figure 2A).

Optical coherence tomography (OCT) is an intravascular imaging method that acquires images at a resolution of ~10 μm, and captures images with an axial resolution 10 times higher than intravascular ultrasound, enabling visualization of the blood vessel wall at near histological resolution [1]. OCT has proven to be a useful modality in coronary angiography, and may have similar applications in evaluating intracranial atherosclerotic disease. OCT is able to detect ulceration, large lipid pools, fresh thrombus, and severe calcification laying on the surface of the vessel. More importantly, stent apposition and expansion, plaque protrusion, and thrombus characteristics can also be clarified through OCT imaging during and post-interventional stenting, which is vital for subsequent therapy.

All patients at our center who undergo stenting are pretreated with aspirin (100 mg/d) and clopidogrel (150 mg followed by 75 mg daily). Neurological assessment was performed before and after the procedure. The patients who undergo OCT sign explicit consent. OCT studies are conducted according to the guidelines of the Declaration of Helsinki, and approved by the Institutional Review Board of Xuanwu Hospital (protocol code (20200913) and date of approval: 30 September 2020. Prior to stenting, standard guidewire placement via a percutaneous puncture of the right femoral artery is performed. A Dragonfly Duo (St. Jude Medical, Little Canada, MN, USA) OCT imaging catheter is placed prior to stenting in order to determine stent sizing, and OCT images in this case confirmed severe stenosis with atherosclerotic plaque formation, as well as fresh mural thrombus (i.e. red thrombus) at the distal end of the stenosis, which has not otherwise been clearly identified. The OCT images demonstrated that this as an irregularly high signal area into the lumen, with hypo-reflective signal attenuation in addition to a fibro-lipid plaque component (Figure 2B–E). A 4.0×12 mm Xience Xpedition (Abbott International, Diegem, Belgium) everolimus-eluting coronary stent was used for left V2 segment stenting. Post-stenting DSA confirmed no residual stenosis (Figure 3A), and OCT was then performed to check for stent apposition, stent expansion, and plaque or thrombus protrusion, which provided excellent visualization of the stent and vessel wall apposition. Good apposition regarding the proximal stenosis was seen, whereas red thrombus attached to the distal end was seen on OCT, suggesting possible future embolization risk (Figure 3B–D). Immediately after intervention, the patient experienced dizziness, confusion, vomiting, and diarrhea, and right cerebellar infarction was confirmed by magnetic resonance imaging (MRI) (Figure 3E–H). This was surmised to be based on the aforementioned red thrombus seen distally, and thus, treatment with a standard dual antiplatelet regimen and statin therapy was prescribed for one year. Six months after treatment, the symptoms improved, and six-minute walking distances were successful with no gait impairment.

The incidence of red thrombus embolism with distal embolization is high, and possesses a considerable mortality rate, but timely diagnosis is uncommon, and is usually arrived upon after a stroke has occurred. Ideally, this diagnosis should be made at the time of intervention if possible to avoid complications. While DSA has been considered an effective method for visualizing thrombus and embolism as filling defects, the structure of the lumen and the composition of thrombi is relatively poorly resolved. By contrast, OCT is an optical imaging technology characterized by real-time, dynamic intraluminal imaging and high resolution, and very much is its nascence within intracranial vasculature. The OCT imaging guidewire can be passed into distal small vessels for imaging, and OCT images have the ability to clearly depict the luminal structure without neurological complications and identify missed thrombi on DSA, as well as distinguish different types of thrombi, which may guide anticoagulant therapy [2]. OCT and other imaging modalities complement each other and may additionally aid in discriminating plaques that are eligible for stenting [3]. To this end, OCT has also shown promise in forensic pathology and radiology, with coronary dissection and rupture visible at virtual autopsy, in addition to micron-level changes in ocular and dermal tissue [4,5].In this case, red thrombus was quite readily confirmed by OCT, as it showed a hyperreflective protrusive mass with an irregular surface, causing marked posterior shadowing, the hallmarks of a large thrombi by this technique [6]. Moreover, representative images of plaque erosion in our patient with symptomatic left vertebral stenosis were also readily seen. Plaque erosion is considered to be one of the sources of embolic complication after stenting, and erosion distal to stent struts just after stent deployment was seen on OCT. This is, to our knowledge, the first report of pre and post-stenting V2 segment assessment with OCT imaging. DSA and OCT both indicated an intact fibrous cap with thrombus formation, illustrating plaque erosion in V2 segment severe stenosis [7]. Understanding the role of judicious use of OCT may improve the diagnosis of red thrombus and plaque erosion in carefully selected patients, and stands to aid in the diagnosis of complex plaque, as well as the anticipation of complications relating to these.

**Figure 1:**
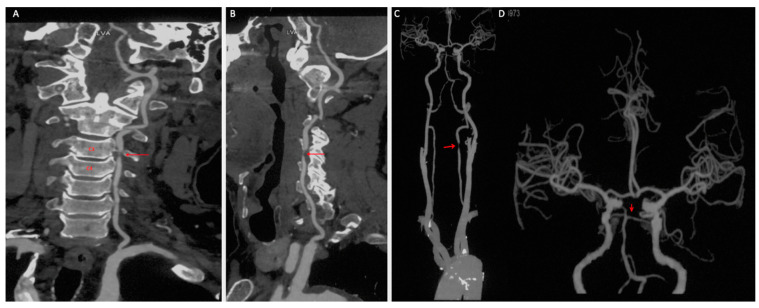
Target vertebral artery anatomy before endovascular intervention. (**A**,**B**) Computed tomography angiography of the left V2 segment before intervention. Dominant left vertebral artery V2 segment severe stenosis ((**A**–**C**), red arrow) and an undeveloped left posterior cerebral artery, together with incomplete circle of Willis, are shown ((**D**), red arrow).

**Figure 2:**
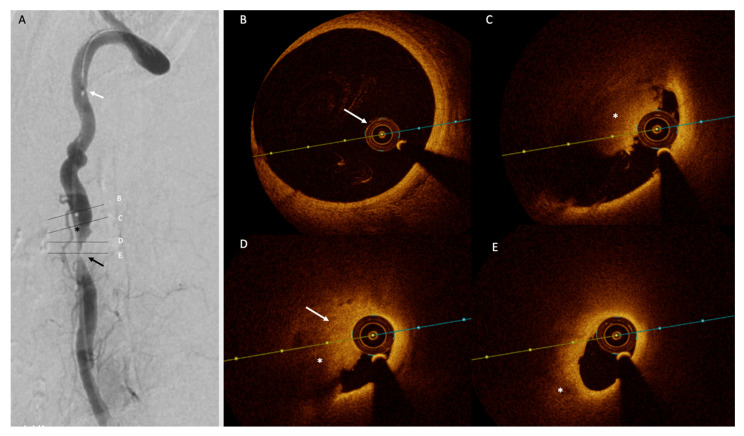
Pre-stenting angiography and OCT images. (**A**) Digital subtraction angiography image of the left V2 segment prior to stenting. Intraluminal thrombus was seen as an irregular intraluminal filling defect (black arrow), and severe stenosis of the underlying vessel was deemed suitable for stenting. An intraluminal filling defect above the stenosis was illustrated by OCT layer (dash). Cross-sectional OCT images during standard pullback with the OCT catheter tip location indicated as a white arrow showed (**B**) normal lumen proximally with no thrombus and (**C**–**D**) red thrombus distally manifested as an irregular hyper-reflective region projecting into the lumen (*), with posterior shadowing. (**E**) Eccentric stenosis relating to the atherosclerotic plaque containing fibro-lipid components appears as a homogenous, hypo-reflective region (*).

**Figure 3:**
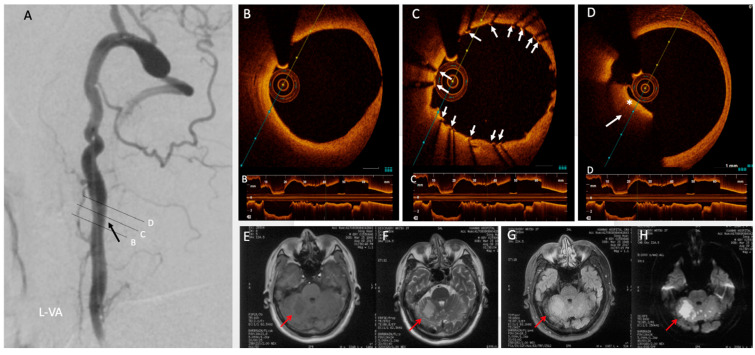
Post-stenting angiography and OCT images and post-procedural MRI. (**A**) DSA of the left V2 segment post-stenting showed marked improvement in previously severe stenosis, well-covered by the stent (black arrow). Cross-sectional OCT images during standard pullback demonstrating (**B**) normal lumen proximally with no thrombus and (**C**) satisfactory stent wall apposition (white arrow), whereas (**D**) distally a hyper-reflective area (*) projecting into the lumen with apparent posterior shadowing indicated red thrombus (white arrow) attached, which had no stent coverage on OCT, suggesting embolization risk. (**E**–**H**) MRI images following intervention when the patient developed clear symptoms demonstrate right cerebellar infarction (red arrow), presumed to be related to red thrombus, which was not covered.

## Data Availability

The data presented in this study are available on request from the corresponding author. The data are not publicly available due to patient privacy.

## Abbreviations

TIA	Transient ischemic attack
CTA	Computed tomography angiography
DSA	Digital subtraction angiography
OCT	Optical coherence tomography
DES	Drug-eluting stent
MRI	Magnetic resonance imaging
